# The Immune System Bridges the Gut Microbiota with Systemic Energy Homeostasis: Focus on TLRs, Mucosal Barrier, and SCFAs

**DOI:** 10.3389/fimmu.2017.01353

**Published:** 2017-10-30

**Authors:** Martina Spiljar, Doron Merkler, Mirko Trajkovski

**Affiliations:** ^1^Faculty of Medicine, Department of Cell Physiology and Metabolism, Centre Médical Universitaire, University of Geneva, Geneva, Switzerland; ^2^Diabetes Center, Faculty of Medicine, Centre Médical Universitaire, University of Geneva, Geneva, Switzerland; ^3^Faculty of Medicine, Department of Pathology and Immunology, Centre Médical Universitaire, University of Geneva, Geneva, Switzerland; ^4^Institute of Genetics and Genomics in Geneva (iGE3), University of Geneva, Geneva, Switzerland

**Keywords:** gut microbiota, immune system, toll-like receptors, short chain fatty acids, mucosal barrier, metabolism, dysbiosis

## Abstract

The gut microbiota is essential for the development and regulation of the immune system and the metabolism of the host. Germ-free animals have altered immunity with increased susceptibility to immunologic diseases and show metabolic alterations. Here, we focus on two of the major immune-mediated microbiota-influenced components that signal far beyond their local environment. First, the activation or suppression of the toll-like receptors (TLRs) by microbial signals can dictate the tone of the immune response, and they are implicated in regulation of the energy homeostasis. Second, we discuss the intestinal mucosal surface is an immunologic component that protects the host from pathogenic invasion, is tightly regulated with regard to its permeability and can influence the systemic energy balance. The short chain fatty acids are a group of molecules that can both modulate the intestinal barrier and escape the gut to influence systemic health. As modulators of the immune response, the microbiota-derived signals influence functions of distant organs and can change susceptibility to metabolic diseases.

## Microbiota Shapes the Immune System and the Host Metabolism

The human microbiota comprises an enormous amount and variety of microorganisms. Among archea, eukarya, and viruses, bacteria are the most abundant inhabitants of the human host. The human gastrointestinal tract is one of the world’s most densely packed microbe habitats (1, 2). By coevolution with humans, a symbiotic relation evolved with the host providing a living environment and nutrients in exchange for support in nutrient degradation and protection from pathogenic microorganisms. The development of 16S rRNA sequencing allowed estimates of the abundance of microbiota in greater detail, revealing the gram-negative Bacteroidetes and gram-positive Firmicutes as the most abundant phyla in the human gut. Changes in the Firmicutes to Bacteroidetes ratio, as inducible among others by high-fat diet (3), can impact metabolism (4–7), and lead to symptoms of type 1 and 2 diabetes, colitis, and obesity (8). Interestingly, transplantation of microbiota from obese human or murine donors to germ-free (GF) mice is sufficient to induce insulin resistance and increased adiposity compared to the lean microbiota transplanted controls (9–12). The Firmicutes and the Bacteroidetes, together with the other inhabitants of the gut, can also modulate immune function (13). The importance of the microbiota for the formation of a fully functional immune system first became evident by studying the GF animals, which are bred and housed in an environment devoid of microorganisms. The immune tissues or local immune cell subsets that are in direct contact with microbiota, as in the gut, are different in GF animals. The gut of the GF mice shows fewer and smaller Payer’s patches (14), an altered mucus layer (15), not fully developed gut-associated lymphoid tissues, and no formation of isolated lymphoid follicles, which help inducing local immune responses (16). Counts of the immune cells residing within the gut are decreased, including the intraepithelial CD8^+^ T cells, the lamina propria CD4^+^ T cells, and their subsets type 17 T helper cells (Th17) and regulatory T cells (Tregs), as well as immunoglobulin G counts (17, 18). The absence of gut microbiota leads to functional alterations in immune and intestinal epithelial cells, which express less microbe sensing toll-like receptors (TLR) (19) and major histocompatibility complex II molecules for antigen presentation (20).

Certain aspects of an underdeveloped, less responsive gut immune system seem to have beneficial effects in obesity. GF, or microbiota depleted mice using antibiotics show improved glucose and insulin tolerance, accompanied by reduced adiposity (9), increased browning of their white fat depots (21), and are protected from diet-induced obesity (DIO) (22–24). The fat browning promotes energy dissipation in the form of heat, enabled by the uncoupling of oxidative phosphorylation from ATP biosynthesis (25). In part, the metabolic effects and the browning phenotype are mediated by the innate immune system and the increased M1 to M2 macrophage polarization that potentiates the fat browning either after cold (26, 27), or after microbiota depletion (21). The GF metabolic phenotype is partially reversible once mice are transplanted with microbiota from donor mice. These observations from GF animals, or from the mice transplanted with microbiota, illustrate that the microbiota is essential in the progression of metabolic imbalances and in the development of a functional immune system. This review will summarize how microbial molecules and changes in the microbiota composition are sensed by the immune system and how microbial products and consequently the immune response can spread into the circulation to cause systemic and metabolic consequences, starting in the gut and moving toward the periphery. We highlight TLR signaling, gut barrier modulation, and short chain fatty acid (SCFA)-mediated interactions as important mechanisms in this process.

## TLRs Sense Microbial Products in the Gut and Periphery to Influence Systemic Immunity and Metabolism

One of the immune system’s tasks is to decide whether a microbe is a harmless commensal or an invading and potentially pathogenic one. As the first line of defense, the innate immune system will recognize both kinds of microbes as they include certain non-self patterns. This is particularly important for ensuring correct intestinal homeostasis (28). One class of molecules that recognizes such patterns is the pattern recognition receptor family that includes the TLRs. There are 10 TLRs in humans and 12 in mice found on the cell or endosomal membranes of different cell types including macrophages, dendritic cells (DCs), and non-immune cells such as epithelial cells, hepatocytes, or adipocytes. Activation of such receptors initiates downstream signaling cascades that often result in induction of cytokine expression. These cytokines can further influence other immune cells and accordingly dictate the tone of an immune response.

**Figure 1:**
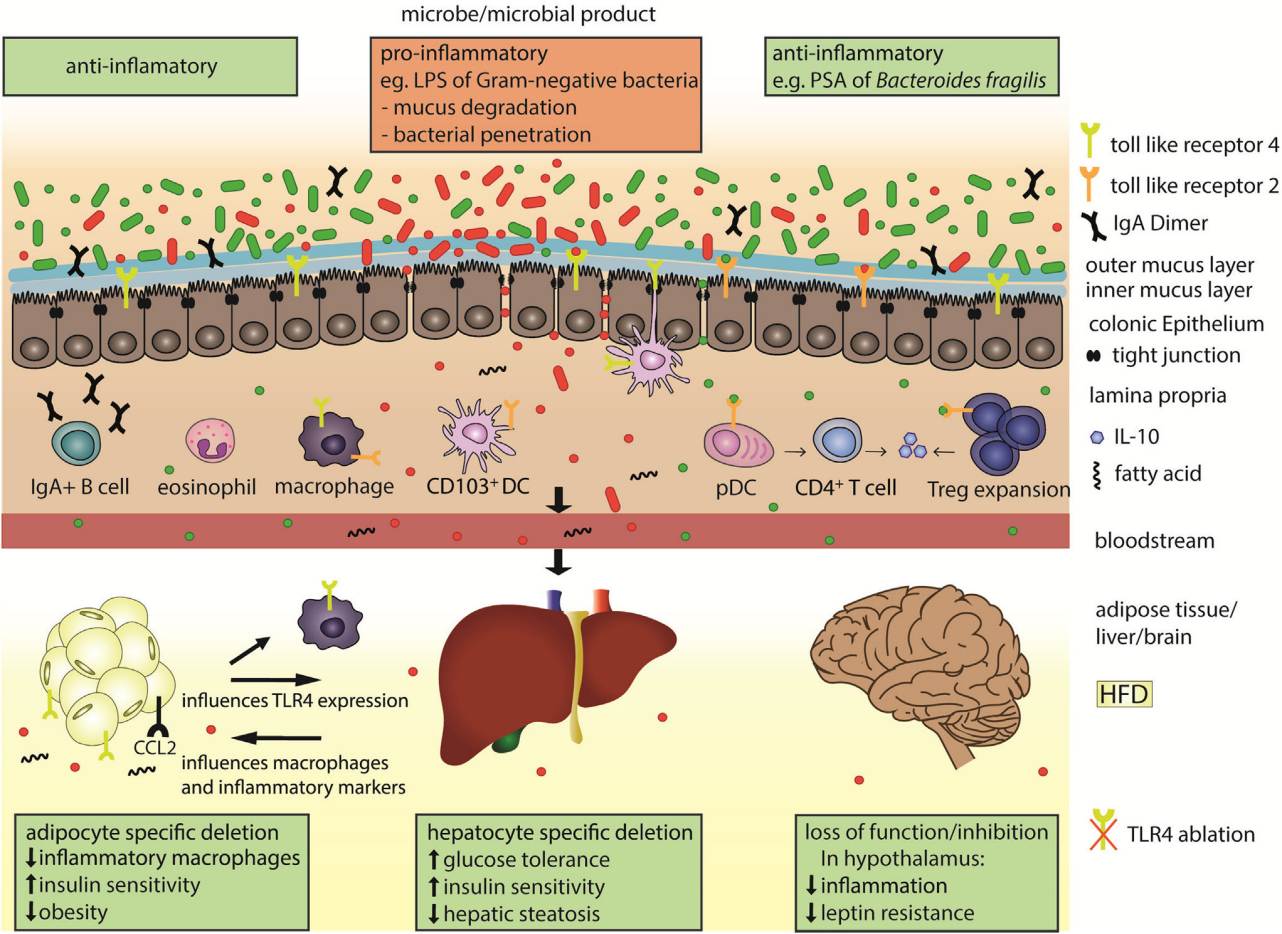
Microbial products are sensed by toll-like receptors (TLRs) in the gut and in the periphery to influence systemic immunity and metabolism. The colonic epithelial barrier is composed of an outer mucus layer that is habitat for microbes and an inner, impenetrable layer, in addition to tight-junction connected epithelial cells. Under normal physiologic conditions and when sufficient anti-inflammatory microbial products (green) are present in the gut, the gut lining is well protected, with plasma B cell secreted Immunoglobulin A (IgA), regulatory T cells (Tregs), and eosinophils. The polysaccharide A (PSA) of *Bacteroides fragilis* is a TLR2 ligand that promotes secretion of the anti-inflammatory cytokine interleukin 10 (IL-10) from the plasmacytoid dendritic cells (pDCs)-activated CD4^+^ T cells, or from the expanded Tregs cells. Under microbial dysbiosis, proinflammatory microbes (red) predominate in the gut and can be sensed, e.g., by TLR4 on CD103^+^ dendritic cells (DCs) or macrophages. Such microbes can degrade and invade the second colonic mucus layer and escape the gut when the epithelial cell lining and tight junctions are disrupted. Subsequently the inflammatory microbial products, like the LPS of gram-negative bacteria, can reach distant organs through circulation. LPS and fatty acids are elevated under high fat diet (HFD) and activate TLR4 signaling. Organ-specific effects after HFD include upregulation of TLR4 and macrophage attractant CCL2 in the adipose tissue, which leads to inflammation and supports obesity.

Toll-like receptor 2 signals as a heterodimer with TLR1 or TLR6 and recognizes a wide variety of signals on fungi and bacteria. One ligand of TLR2 is polysaccharide A (PSA) from *Bacteroides fragilis* that induces anti-inflammatory responses, activates plasmacytoid DCs, interleukin 10 (IL-10) production of CD4^+^ T cells, promotes clonal expansion and induction of Treg cells, and suppresses Th17 production in the gut (Figure 1) (29–31). These aspects potentially contribute to the amelioration of inflammation in animal models such as experimental autoimmune encephalomyelitis and colitis after PSA administration (29, 32, 33) or after colonization of GF mice with Bacteroidetes (34). Anti-inflammatory responses upon TLR2 activation were suggested as a way to recognize commensals as non-pathogenic. This suggests that specific microbial-derived mechanisms actively promote immunologic tolerance to symbiotic bacteria. In contrast, TLR2 signaling can also result in proinflammatory responses, as for instance upon detection of *Lactobacillus plantarum* teichoic acid d-alanylation (35). Although the highest encounter opportunity between microbes and immune cells is the gut, microbial products are also detected by TLRs at peripheral sites, influencing systemic and metabolic effects. *Tlr2* expression is increased in visceral adipose tissue in mice fed a high fat diet (HFD) compared to a normal chow diet, resulting in tumor necrosis factor alpha (TNF-α) expression, thereby supporting a low grade inflammation in tissues (Table 1) that is typical of obesity (36). *Tlr2* inhibition or ablation results in improved insulin sensitivity and decreased inflammation and adiposity in a DIO mouse model (37–39). These findings support the role of TLR2 as a proinflammatory mediator of metabolic symptoms.

**Table 1:** TLRs and SCFA bridge microbiota with host metabolism as initiators of immunologic processes.

	Target	Effect	Reference
TLRs			
**TLR2**		Insulin resistance, inflammation	(37–39)

TLR4	Adipose tissue	Inflammation, macrophage accumulation	(40–42)
	Hypothalamus	Inflammation, leptin resistance	(43)

TLR5	Liver	Protection from liver disease	(44)

**SCFAs**			

SCFAs	Intestine	Gut-derived hormone secretion, gluconeogenesis	(45–47)
	Intestine	DIO, insulin resistance	(48, 49)
	Lymphoid tissues	Gut barrier, Tregs	(50)
		Autoreactive T cells (T1D)	

Acetate	Adipose tissue	Fat accumulation, insulin signaling, adipose tissue browning, lipid storage	(51–53)
	Liver	Hepatic function	(52)
	Various	Energy expenditure, insulin secretion, ghrelin secretion, hyperphagia	(51, 53)

Butyrate	Intestine	M2 macrophage polarization, colonic mucus secretion	(54, 55)

Proprionate	Intestine	Gut hormones	(49)
	Brain	Food intake	

*TLR and SCFA signaling occurs in response to microbiota changes in the intestine and can reach distant organs, influencing the inflammatory and metabolic state to initiate systemic effects*.

*DIO, diet-induced obesity; HFD, high-fat diet; TLR, toll-like receptor; Tregs, regulatory T cells; T1D, type 1 diabetes*.

Toll-like receptor 4 mainly recognizes the Gram-negative bacterial cell wall component lipopolysaccharide (LPS). LPS levels in blood are increased in obesity or after high caloric diet (HCD) feeding (56) and are associated with increased Firmicutes to Bacteroidetes ratios. TLR4 signals through the MyD88-dependent pathway, or by TIR-domain-containing adapter-inducing interferon-β regulation of type 1 interferons *via* interferon regulatory factor 3 (IRF3). *Tlr4* expression is increased in adipose tissues, peripheral blood or muscle of obese or type 2 diabetes patients (57, 58) and in adipose tissues of obese *db/db* mice (59) and correlates with insulin resistance. LPS induces adipose tissue inflammation through TLR4 (Figure 1), and increases *Ccl2* expression on adipocytes. This chemokine contributes to a microbiota-induced macrophage accumulation and WAT inflammation in lard-fed mice (40). Loss of function mutation in *Tlr4*, or TLR4 deletion reduces macrophage infiltration into the adipose tissue, promotes their anti-inflammatory M2 polarization (41), decreases tissue and circulating inflammatory marker levels, and diminishes inflammation in the streptozotocin-induced mouse model for type 1 diabetes (42). *Irf3* knockdown improves insulin sensitivity, mediates an anti-inflammatory phenotype, and also increases white fat browning (60). Intestinal epithelial cell-specific *MyD88* deletion decreases fat mass accumulation, body weight gain and glucose intolerance in diet-induced obese mice (61). Apart from LPS, fatty acids can also increase TLR4 signaling in several cell types including adipocytes and macrophages (62) and promote visceral obesity and insulin resistance (63). Interestingly, adipocyte-specific *Tlr4* KO mice demonstrate two distinct effects of TLR4 on the adipose tissue. Specifically, these mice show increased whole body and muscle insulin resistance after HFD feeding, but also improved insulin sensitivity after an acute lipid challenge during a hyperinsulinemic euglycemic clamp (64). Adipocyte-specific *Tlr4* deletion also modifies *Tlr4* expression in other tissues, as it decreases *Tlr4* expression in peritoneal macrophages and liver. Alteration of TLR levels in the liver has important systemic metabolic effects. Hepatocyte-specific *Tlr4* deletion improves glucose tolerance, insulin sensitivity, and hepatic steatosis in HFD fed obese mice (65). In addition to the local signaling in the gut or the peripheral tissues, the TLR-mediated effects also reach the brain. TLR4 inhibition decreases inflammation and leptin resistance in the hypothalamus (43). Deletion of the TLR downstream signaling molecule *MyD88* in the central nervous system prevents obesity and leptin resistance (66). Furthermore, in older or HCD-fed mice, *Tlr4* expression was increased in the pro-opiomelanocortin neurons from the arcuate nucleus of the hypothalamus, a central metabolism regulating brain area (67).

Toll-like receptor 5 recognizes bacterial flagellin, a component of the bacterial locomotion system. This signaling pathway causes an anti-inflammatory response by inducing interleukin 1 receptor antagonist secretion and diminishing IL-1β and inflammasome activity (68). *Tlr5* KO mice were first reported to be prone to develop metabolic syndrome, including insulin resistance and increased adiposity, which correlated with changes in their microbiota composition (69). This phenotype was also confirmed in an epithelial cell-specific *Tlr5* KO (70) and was associated with low-grade inflammation. Interestingly, these initial findings could not be reproduced in the same *Tlr5* KO mouse line by a different research group (71, 72), nor in a new *Tlr5* KO model (73). While a clear explanation for this discrepancy is missing, a possible reason could lay in the differences in the microbiota composition between different mouse facilities, and/or may depend on whether initially pro- or anti-inflammatory molecules are predominant in the local gut environment. Specific *Tlr5* deletion in hepatocytes confers predisposition to diet-induced liver pathology. This is accompanied by elevated expression of proinflammatory cytokines and dependent on the Nod-like-receptor C4 inflammasome and rescued by microbiota depletion (44). Similarly, hepatocyte-specific deletion of *MyD88* leads to inflammation, glucose intolerance, and hepatic insulin resistance, accompanied by alterations in the microbiota composition (74). These observations suggest that hepatocyte TLR5 plays a role in protecting the liver and in preventing diet-induced liver disease.

## Dysregulation of Gut Mucosal Surfaces Can Contribute to Systemic Microbial Effects

Changes in the gut microbiota can modulate systemic microbe-derived metabolite levels by affecting their biosynthesis, or by changing the intestinal permeability and gut barrier. The mucosal surfaces are another first line defense component of the innate immune system, at which most of the microbe–host interactions take place. In the gastrointestinal tract, the mucosal surface consists of an epithelial cell layer, in which the mucus-secreting goblet cells are responsible for forming a second protective barrier, the mucus layer. Due to its physiologic function in the gut for food absorption, the mucosal surface is naturally a thin and permeable layer. The protection against microbial invaders under natural physiologic conditions is ensured by several mechanisms, including tight junctions between the epithelial cells, which are constantly renewed from stem cells allowing immediate repair in case of cell loss or damage. Mucus is composed of charged glycoproteins called mucins and its density and stickiness traps microbes and their products, preventing contact to the epithelial cells. Within the large intestinal mucus, there are two layers, a loose outer layer that is a natural habitat for microbes and a dense inner layer, impermeable for bacteria (15).

Dysregulation of the mucus structure by changes in its thickness or penetrability can lead to inflammation. This is evident in the *mucin 2* (*Muc2*) KO mice where microbiota is in direct contact with the epithelial cells (75, 76). TLRs were shown to mediate MUC2 secretion (77) and were implied in epithelial homeostasis (28, 78). *MyD88* deletion in intestinal epithelial cells leads to defects in mucosal barrier functions with reduced *Muc2* mRNA expression and increases epithelial cell penetration by bacteria (79, 80). GF mice have easily penetrable mucus in the large intestine, whereas conventionally raised mice show a thick, impenetrable mucus layer (15, 81). The level of penetration and degradation depends largely on the gut microbiota composition (82). Although most bacteria metabolize non-digested food polysaccharides (83, 84), many bacteria can use mucin glycans as an energy source (85–87). This substantial role of the microbiota also indicates that an imbalance in the bacterial species composition can influence the protective capacity of the mucus barrier. This kind of dysbiosis can result from a variety of different factors, including a decreased bacterial diversity, lack of secretory antibodies that line the mucus layer, and lack of Tregs (88) or eosinophils (89, 90). Once the mucus barrier is invaded and microbes are in closer contact to epithelial cells, mucus production and stem cell number is increased in the intact epithelium, and this is triggered by MyD88 signaling (91). This illustrates that the epithelium counteracts the potential threat of microbes invading the mucus layer. However, similar to the mucus layer, the epithelial cell lining can also become permeable. Damage to the epithelial layer can originate from altered tight junction composition or epithelial cell stress (92). Autophagy, a process in which a phagosome is formed in the cytoplasm to engulf various contents, is also important for maintaining an intact epithelial barrier and in the regulation of mucus secretion (93, 94). Disruption of autophagy induced by coding polymorphism (Thr300Ala) in the autophagy-related 16-like 1 leads to decreased antibacterial autophagy, induces epithelial cell stress, and allows systemic penetration of bacteria and inflammation (95).

Gut permeability is increased with HFD, in obesity and in diabetes (96). HFD-induced nonalcoholic fatty liver disease (NAFLD) at thermoneutral housing conditions (30°C) is associated with an altered microbiome, increased intestinal permeability and induction of proinflammatory responses that are active in this disease in humans. Depletion of hematopoietic *Tlr4* or gram-negative microbiota leads to altered immune responsiveness and protects from NAFLD at thermoneutrality (97). With dietary fiber deficiency, a subset of mucin-degrading bacteria increases, which express mucin-degrading carbohydrate-active enzymes (CAZymes) that enable access to host-secreted mucus glycoproteins as a nutrient (98), resulting in the degradation of the colonic mucus barrier. This increases LPS levels systemically. Glucagon like peptide (GLP)-1 and 2 levels, which have beneficial metabolic effects (99) and could affect gastric barrier function (100), can be regulated by LPS. In addition, obese mice have impaired IL-22 induction from innate lymphoid and CD4^+^ T cells under immune challenge, and IL-22 reverses the HFD-induced epithelial cell stress (92). IL-22 depletion results in defects in mucosal defense and metabolic symptoms, many of which can also be reversed by exogenous IL-22 (101).

A group of molecules that can both modulate the intestinal barrier and escape the gut to influence systemic health are the SCFAs. SCFAs acetate, butyrate, and propionate are produced by bacterial anaerobic metabolism of indigestible dietary components, including fiber. They signal *via* G-protein-coupled-receptors such as GPR41, GPR43, and GPR109a (102, 103) and are important regulators of gut homeostasis and epithelial barrier maintenance. Acetate enhances protection against infection (104), while butyrate is an energy source for colonocytes (105–107) and can regulate stem cell proliferation and anti-inflammatory macrophage polarization (54, 108) with its histone deacetylase inhibiting function. Butyrate also increases colonic mucus secretion, both directly by promoting *Muc2* and glycoltransferase expression (55, 109), and indirectly by promoting autophagy (93, 94, 110, 111). Further protection against inflammation through SCFAs is accomplished by their regulation of Tregs (112). The elevated circulating SCFA levels in mice fed a high-fiber diet result in allergy protection by impaired Th2 differentiation and increased phagocytosis by DCs, while low-fiber diet promotes allergic inflammation (113). In type 1 diabetes, the SCFA amount correlates with improvement of symptoms *via* limitation of autoreactive T cells, induction of Tregs, and enhanced gut barrier (50). The beneficial SCFA effects (52, 114) can act through local induction of gut-derived hormone secretion, including GLP-1 and peptide YY (45–47), and through the circulation (115). SCFA-mediated beneficial metabolic effects on health can be mediated by induction of intestinal gluconeogenesis (48). Specifically, supplementation with acetate in drinking water or through nanoparticles, butyrate gavage alone or combined with propionate, or adding propionate, butyrate, and acetate to the diet, improve host physiology and glucose metabolism (49, 51, 52, 116) (Table 1), which in the case of propionate seems to be mediated by vagus-nerve stimulation by the peripheral nervous system (48). Conversely, the microbiota-mediated increase in acetate turnover that occurs during exposure to a HFD diet might mediate a feedback loop between the gut microbiota and the parasympathetic nervous system, promoting hyperphagia owing to increased ghrelin secretion, and increased energy storage (53). The site of stimulation seem to be important for the outcome of SCFA-mediated effects (117, 118) and points to the need for further exploration of the general role of SCFAs in regulating obesity.

## Conclusion

The encounter of microbial molecules by TLRs initially provokes a local immune response in the gut. Such local reactions can spread by escape of the microbial products from the gut, which can then reach distant organs. In addition to immune cells, the adipose tissue, liver, and brain are important TLR-expressing targets and are all potent metabolic regulators with systemic effects. The escape of microbial factors from the gut can result from perturbations in the gut microbiota and its interaction with immune system components that weakens the mucosal and epithelial lining of the gut. When the integrity of the mucus layer and intestinal epithelium is impaired, harmless commensals can become a threat, crossing the epithelium to invade the bloodstream, causing systemic infection or inflammation, which favors development of immune-mediated and metabolic diseases. Consequently, peripheral anti- or proinflammatory responses take place and modulate host metabolism, emphasizing to which substantial extent microbiota can influence systemic health.

In addition to the above discussed mechanisms, bile acids, which can be modified by microbiota or result from microbiota changes, could modulate host metabolism (119, 120) and also have immunomodulatory effects (121). Further gut microbiota derived metabolites, including tryptophan, phenylalanine, tyrosine, and polyamines, can regulate the immune system and the host metabolism with potential effects on health. For instance, the molecular signature of such interactions is described for the regulation of mucosal integrity and inflammatory signaling (122–124) by bacterial indoles binding to the pregnane X receptor (125) *via* pathways involving TLR4 (126) and NFkB. Indoles (127–132) and dietary ligands such as flavonoids (133) can be sensed by aryl hydrocarbon receptor nuclear translocator 2, which modulates epithelial barrier integrity (134–139) and provokes immune changes (140–144). Further immunologic components that can bridge microbiota with host metabolism include the nucleotide-binding oligomerization domain-like receptors (NOD-like receptors) and innate lymphoid cells that have been reviewed elsewhere (145). While linking host metabolism with microbiota alterations is a challenging task, the integrative metabolomics–metagenomics approaches are a promising way to provide a better understanding of these interactions. For example, using such approaches Pedersen and colleagues identified *Prevotella copri* and *Bacteroides vulgatus* as the main species driving the association between biosynthesis of branched-chain amino acids and insulin resistance, where *P. copri* increased their circulating levels, and induced insulin resistance and glucose intolerance (146).

Reprogramming of microbiota in patients can be an accessible and promising anti-obesity treatment. Thus, understanding the complex and reciprocal interplay between the microbiota and the immune system, and how this relationship can modulate metabolic parameters could result in advances toward the treatment of metabolic diseases.

## Author Contributions

The authors reviewed literature and conceived the manuscript. MS wrote the manuscript and prepared the figure. DM provided insightful comments and corrections. MT wrote paragraphs, corrected the manuscript, and provided guidance.

## Conflict of Interest Statement

The authors declare that the research was conducted in the absence of any commercial or financial relationships that could be construed as a potential conflict of interest.
